# Discovering the Protective Effects of Resveratrol on Aflatoxin B1-Induced Toxicity: A Whole Transcriptomic Study in a Bovine Hepatocyte Cell Line

**DOI:** 10.3390/antiox10081225

**Published:** 2021-07-29

**Authors:** Marianna Pauletto, Mery Giantin, Roberta Tolosi, Irene Bassan, Andrea Barbarossa, Anna Zaghini, Mauro Dacasto

**Affiliations:** 1 Department of Comparative Biomedicine and Food Science, Division of Pharmacology and Toxicology, University of Padova, Viale dell’Università 16, Legnaro, 35020 Padova, Italy; marianna.pauletto@unipd.it (M.P.); mery.giantin@unipd.it (M.G.); roberta.tolosi@phd.unipd.it (R.T.); irene.bassan@unipd.it (I.B.); 2 Department of Veterinary Medical Sciences, University of Bologna, Via Tolara di Sopra 50, Ozzano dell’Emilia, 40064 Bologna, Italy; andrea.barbarossa@unibo.it (A.B.); anna.zaghini@unibo.it (A.Z.)

**Keywords:** aflatoxin B1, aflatoxicosis, mycotoxins, resveratrol, antioxidants, cattle, liver, RNAseq, transcriptome, cancer

## Abstract

Aflatoxin B1 (AFB1) is a natural feed and food contaminant classified as a group I carcinogen for humans. In the dairy industry, AFB1 and its derivative, AFM1, are of concern for the related economic losses and their possible presence in milk and dairy food products. Among its toxic effects, AFB1 can cause oxidative stress. Thus, dietary supplementation with natural antioxidants has been considered among the strategies to mitigate AFB1 presence and its toxicity. Here, the protective role of resveratrol (R) has been investigated in a foetal bovine hepatocyte cell line (BFH12) exposed to AFB1, by measuring cytotoxicity, transcriptional changes (RNA sequencing), and targeted post-transcriptional modifications (lipid peroxidation, NQO1 and CYP3A enzymatic activity). Resveratrol reversed the AFB1-dependent cytotoxicity. As for gene expression, when administered alone, R induced neglectable changes in BFH12 cells. Conversely, when comparing AFB1-exposed cells with those co-incubated with R+AFB1, greater transcriptional variations were observed (i.e., 840 DEGs). Functional analyses revealed that several significant genes were involved in lipid biosynthesis, response to external stimulus, drug metabolism, and inflammatory response. As for NQO1 and CYP3A activities and lipid peroxidation, R significantly reverted variations induced by AFB1, mostly corroborating and/or completing transcriptional data. Outcomes of the present study provide new knowledge about key molecular mechanisms involved in R antioxidant-mediated protection against AFB1 toxicity.

## 1. Introduction

Aflatoxins (AFs) are mycotoxins naturally affecting cereal grains and animal feeds around the world [1,2]. They can cause various diseases and health issues in humans and farmed animals. In the latter, AFs exposure might have severe implications, such as liver damage, nutritional or immunological consequences. These effects lead to decreased animal growth and productivity [3,4,5], ultimately resulting in significant economic losses [6,7]. The most toxic member of this group of mycotoxins is aflatoxin B1 (AFB1), that is also known as the most potent natural hepatocarcinogen [8], thus leading the International Agency for Research on Cancer (IARC) to classify this mycotoxin as a Group I human carcinogen [9].

The great toxicity of AFB1 is based on a bioactivation process. Indeed, AFB1 is biologically activated by a number of cytochromes P450 (CYPs), namely CYP1A and CYP3A, to an extremely reactive and electrophilic derivative, AFB1-8,9-epoxide (AFBO), that binds DNA and proteins [10]. Such AFBO-DNA and AFBO-protein adducts inhibit RNA and protein synthesis [3], ultimately leading to severe toxicity and eventually cancer development [8].

Besides AFB1, the most toxic aflatoxin is AFM1. AFB1, following animal intake, can be rapidly hydroxylated to form AFM1, which is efficiently transferred to milk, thus representing a safety concern for humans consuming dairy products [11]. Aflatoxin B1 can be also converted to relatively less-toxic metabolites, such as aflatoxicol (AFL). 

Remarkably, species differences in the constitutive expression and catalytic activity of bioactivating and detoxifying enzymes appear crucial in determining the different susceptibility to AFB1 among farmed species [12]. In this respect, the presence of rumen microflora in cattle is a key factor in mitigating AFB1 toxicity, mainly because it converts part of the ingested mycotoxin into less toxic, or nontoxic, metabolites [13]. Nonetheless, in cattle, AFs interfere with the overall ruminal digestive capacity [14], are absorbed at the intestinal level, and are responsible for a plethora of clinical symptoms (e.g., impaired reproductive efficiency, diarrhoea, mastitis) [15,16].

The animal intake of AFs always accompanies the production of Reactive Oxygen Species (ROS) and the resulting oxidative damage [17,18,19], which may be a major trigger of detrimental outcomes [20]. For this reason, to prevent or mitigate aflatoxicosis in farmed animals, the use of feed additives, consisting of botanical extracts with known antioxidant capacities, is a viable option. For instance, the efficacy of curcumin has been widely tested [21,22,23,24,25]; besides curcumin, several natural polyphenols have also been investigated, providing encouraging results [26,27,28,29,30,31,32]. Among them, the use of resveratrol (R) represents a promising strategy.

Resveratrol (trans-3,5,40-trihydroxystilbene) is a polyphenolic phytoalexin naturally found in grapes, red wine, berries, peanut skins, and other plant parts and products. It shows a broad range of pharmacological activities, and in plants it serves as a sort of antitoxin. Being an antioxidant polyphenol, R also shows numerous beneficial health effects, such as anticancer, anti-ageing, and anti-inflammatory effects [33,34,35,36,37,38]. Moreover, R has also been demonstrated to affect lipid metabolism, cell proliferation, differentiation, and apoptosis [39,40,41]. In farm animals, R-supplemented feed has been already tested with some positive results (e.g., in broilers [32,42,43] and swine [44,45]), thus, suggesting that this approach can be effectively implemented in farming practice. Moreover, recent advances in the extraction of R from grape pomace or *Polygonum cuspidatum* [46,47,48] lead us to believe that potential low-cost green processing for the fortification of feed products with this antioxidant might be soon introduced. 

The molecular mechanisms triggered by R have been investigated in farm animal species both in vitro and in vivo. For instance, a whole transcriptome study conducted in bovine skeletal muscle cells has recently demonstrated how R significantly affects the mRNA expression of genes regulating cholesterol and fatty acid metabolism, cell cycle and proliferation [49]. In the same study, sirtuins (SIRTs), regulatory proteins known to protect from inflammation, suppress cell death and activate antioxidant genes [50,51,52], appeared to be differentially regulated by R to a significant extent. Resveratrol supplementation in the washing and fertilisation medium of bull sperm significantly decreased ROS and malondialdehyde (MDA) production, and protected mitochondrial function and acrosomal integrity [53]. In cultured bovine mammary epithelial cells (MAC-T), the nuclear factor erythroid 2-related factor 2 (NRF2), a regulator of antioxidant proteins, is required for the cytoprotective effects of R [54]. In juvenile tilapia fed with an R-supplemented diet the resistance to metabolic dysfunction was improved, and genes associated either with the antioxidant response, e.g., superoxide dismutase (SOD), catalase (CAT), and glutathione peroxidase (GPX), than apoptosis and DNA damage were significantly increased [55]. Maternal dietary R alleviated weaning-associated intestinal inflammation and diarrhoea in offspring through the involvement of biological pathways such as the “T-cell receptor”, “Ras signalling” and “cytokine-cytokine receptor interaction” [56]. In broilers, dietary R increased muscle glycogen content and total SOD and GPX activities, while it decreased muscle MDA content and lactate dehydrogenase (LDH) activity [57].

Recently, the potential anti-AFB1 activity of R has been explored in rodents. In alcohol-AFB1-induced rat hepatocellular carcinoma, a two-week treatment with R (100 mg/kg bw) restored the level of key antioxidant enzymes (i.e., SIRT1, CAT, and GPX) toward normal levels [30,58]. In mice, R decreased AFB1-induced ROS accumulation, ameliorated adverse hepatic functions by increasing hepatic antioxidant capacity and inhibiting the expression of cleaved-caspase-3 protein [59]. Likewise, in Sprague Dawley rats fed AFB1 (7.5 μg/200 g), R effectively prevented the AFB1-induced testicular damage and lipid peroxidation [60]. Conversely, in a study conducted in 2010 in rats, curcumin, but not R, protected from AFB1-induced liver injury [61].

As far as farm animals are concerned, while the overall benefits of dietary R have been largely investigated, the ability of this polyphenol to prevent or mitigate AFB1 toxicity and the underneath molecular mechanisms have been scarcely studied, except for broilers in vivo [32] and cattle in vitro [26,31]. Studies conducted in cattle assessed the effects of R in mammary epithelial cells exposed to AFB1, but hepatic tissue or liver cells have never been subject of investigation. Therefore, the objective of the present study was to investigate the protective role of R against AFB1-induced toxicity in the bovine foetal hepatocyte-derived cell line (BFH12). Moreover, we aimed to elucidate the whole transcriptional changes (RNA-seq) triggered by this natural polyphenol when administered in combination with AFB1. To reach such a goal, cells were at first pre-treated for 16 h with R; then, monolayers were exposed to 3.6 μM AFB1 for further 48 h in combination with R. As a control, cells exposed only to R were also included in the experiment. 

To date, the impact of R on AFB1-mediated whole transcriptional changes in an established hepatic cell line isolated from veterinary species has never been investigated. This study will help in developing R-supplemented diets to mitigate toxicity induced by AFB1 in dairy cattle. Such feeding improvements might contribute to reduce the risk of dairy products contamination, ultimately preventing significant economic losses.

## 2. Materials and Methods

### 2.1. Cell Culture and Chemicals

The bovine SV40 large T-antigen-transduced foetal hepatocyte-derived cell line BFH12 [62] was cultured using the experimental procedures detailed in our previously published paper [63]. Resveratrol (≥99% purity) was obtained from Sigma-Aldrich (St. Louis, MO, USA). All chemicals used in the study are commercially available and of molecular biology grade (see details in [63]). The chemical structure of all the key molecules targeted in the present study (i.e., R, AFB1, AFM1, AFL) has been provided in Figure S1. 

### 2.2. Resveratrol Cytotoxicity

Experimental conditions for the cytotoxicity test were those reported in our previous study [21]. Briefly, four days after seeding, cells were exposed to increasing concentrations of R for a total of 64 h (16 + 48 h; range 5–250 μM). Then, we assessed the ability of R (10, 20, or 30 μM) to mitigate AFB1-induced cells mortality. In accordance with the approach we followed in our previous studies [21,63], BFH12 cells were pre-treated with an aryl hydrocarbon receptor (AHR) agonist, i.e., PCB126. The rationale of this choice lays on the hypothesis that metabolic competence of foetal hepatocytes may be lower compared to that of adult liver cells. Thus, PCB126 pre-treatment was intended to increase the cell responsiveness to AFB1.

Resveratrol concentrations were chosen based on the half maximal inhibitory concentration (IC_50_) obtained in the present study: the selected concentrations were always below the corresponding IC_50_.

### 2.3. Cells Incubation for Gene Expression Analysis

To assess the effects of R treatment upon AFB1-induced transcriptional changes, four independent cell culture experiments were conducted. Details about the experimental procedures (i.e., cells incubation, RNA isolation, RNA concentration and quality assessment) are available in our previously published manuscripts [21,63].

As far as the RNA quality, all samples had an RNA Integrity Number (RIN) value > 7.

### 2.4. Quantitative Real-Time PCR

A targeted qPCR approach was preliminarily performed to assess the effects of R at the selected sub-cytotoxic concentrations (i.e., 10, 20, and 30 μM). Only transcripts known to be regulated by AFB1 (i.e., genes involved in AFB1 metabolism and antioxidant response) were considered.

The target gene expression analysis was carried out on four biological replicates (i.e., independent cell culture experiments). A total of four experimental conditions were assessed: CTRL, R (10, 20, and 30 μM), AFB1, R (10, 20, and 30 μM) + AFB1. Reverse transcription, qPCR amplification conditions, target genes, primers, and qPCR data analysis were described elsewhere [63]. 

### 2.5. RNA-Seq Library Preparation and Sequencing

Based on the preliminary qPCR results, besides CTRL and AFB1 experimental conditions, only cells exposed to the maximum concentration of R (i.e., 30 μM), either alone (R) or in combination with AFB1 (R+AFB1), were subjected to deep transcriptome investigations.

For each experimental condition, three independent biological replicates (i.e., independent cell culture experiments) were assessed. A total of 12 tagged RNA-seq libraries were prepared and sequenced following a 50 bp single-end strategy in an Illumina Hi-Seq 4000 instrument (Fasteris SA, Geneva, Switzerland). Libraries were prepared as previously reported [63]. Briefly, we used the Agilent’s SureSelect Strand Specific RNA Library Preparation Kit (Agilent Technologies, Santa Clara, CA, USA) following the manufacturer’s instruction. Poly(A) mRNA was purified starting from 400 ng of total RNA and chemically fragmented. First-strand and second-strand cDNA were synthetized and end-repaired. Then, cDNA 3′ ends were adenylated and adaptors were ligated. Twelve cycles of PCR were used to amplify the adaptor-ligated cDNA library. The PCR products were then purified and size-selected using the SPRIselect reagent kit (Beckman Coulter, Brea, CA, USA). Library concentrations were measured adopting two approaches: Qubit RNA Assay kit (Life Technologies, Carlsbad, CA, USA), in a Qubit 2.0 Fluorometer (Life Technologies); PCR-based method using the NEBNext Library Quant Kit for Illumina (New England Biolabs, Ipswich, MA, USA). Individual libraries were monitored for insert size using the Agilent DNA 1000 assay kit (Agilent Technologies) on the Agilent Bioanalyzer 2100 instrument (Agilent Technologies).

Notably, libraries representing CTRL and AFB1 conditions have been previously analysed in a stand-alone study assessing the whole transcriptional effects of PCB126 and AFB1 on BFH12 cells [63]. In the present study, these libraries have been normalized and analysed again in the context of a larger dataset including new data (i.e., cells treated with R and R+AFB1).

### 2.6. Differential Expression Analysis

Differential expression (DE) analysis has been conducted as previously described in [21] with minor modifications. Analysis of differential gene expression was conducted using EdgeR [64] and grouping samples according to treatment (i.e., CTRL, R, AFB1, R+AFB1). Pair-wise analysis were performed to assess the transcriptional changes induced by R alone (i.e., R vs. CTRL) and R in combination with AFB1 (i.e., R+AFB1 vs. AFB1). Common and tag-wise dispersions were estimated (*estimateDisp*), a linear model was fitted (*glmQLFit*), and the differential expressed genes (DEGs) were determined (*glmQLFTest*) by setting the following thresholds of significance: FDR ≤ 0.05 and Fold Change (FC) ≥ 1.5 (i.e., log_2_FC ≥ 0.6). The complete R code used for the DE analysis is reported in File S1.

### 2.7. Functional Enrichment Analysis

A functional interpretation of significant DEGs was obtained through GO (Gene Ontology) and KEGG (Kyoto Encyclopedia of Genes and Genomes) over-representation tests implemented in R environment, and using functions included in the *ClusterProfiler* package (i.e., *enrichGO*, *enrichKEGG*) [65]. Ensembl gene identifiers were used to establish two different gene lists (i.e., significantly up- and downregulated genes) and a “background” (i.e., all expressed genes). Dotplots and gene-concept networks were also constructed by using specific functions available in the *ClusterProfiler* package. Gene Ontology terms redundancy was removed by using the *simplify* function (similarity cutoff = 0.5). Dotplots display the most significant enriched terms (*p*-value ≤ 0.05), while gene-concept networks highlight which genes were involved in the significant GO terms.

A pre-ranked KEGG Gene Set Enrichment Analysis (GSEA) [66] was also performed. This statistical approach is helpful to determine whether gene sets defined a priori show statistically significant enrichment at either end of the ranking. A statistically significant enrichment value (Benjamini–Hochberg adjusted *p*-value ≤ 0.05) indicates that the biological activity (e.g., the biomolecular pathway), characterized by the gene set, is correlated with the supplied ranking. The ranked input was prepared as specified in [21]. The analysis was carried out by using the *gseKEGG* function provided by the *ClusterProfiler* package [65].

The detailed R codes used for the bioinformatic analysis are reported in File S1.

### 2.8. Analytical Investigations

At the end of the experiment, medium and cell samples were collected and total AFB1, AFM1, and AFL were measured by LC-MS/MS in all experimental conditions, following the procedure detailed in [63].

### 2.9. Oxidative Stress

Pro-oxidant effects of AFB1 and the potential benefits of R were assessed by measuring lipid peroxidation (MDA production) in the same experimental groups assessed by RNA-seq. Notably, lipid peroxidation is one of the main manifestations of oxidative damage and it is known to play an important role in AFB1 toxicity. The detailed protocol is provided in [21].

### 2.10. NQO1 and CYP3A Enzymatic Activity

The catalytic activity of two key enzymes involved in the antioxidant response and in AFB1 bioactivation, i.e., NQO1 (NAD(H):quinone oxidoreductase 1) and CYP3A, were investigated, as they were here reported to be transcriptionally modulated by treatments. Enzymatic activities were measured in the same experimental groups considered for RNA-seq analysis.

Diaphorase, or NQO1, was selected as it is a multifunctional and stress-inducible protein involved in antioxidant defence and detoxification processes. It reduces quinone to hydroquinone, thus avoiding the formation of semiquinones and species with reactive oxygen radicals that are deleterious to cells [67]. The diaphorase activity was assessed using an Abcam commercial kit (ab184867 NQO1 activity assay kit), following the manufacturer’s instructions.

The cytochrome P450 3A was selected as it plays a pivotal role in the bioactivation of AFB1 and in the formation of the highly reactive epoxide intermediate AFB(1)-8,9-epoxide [8]. Catalytic activity of CYP3A was measured using a Promega commercial kit (P450-Glo^TM^ CYP3A4 assay with Luciferin-IPA), following the protocol detailed in [21]. Six independent cell culture experiments were performed in sextuplicate. Catalytic activity results were expressed as normalized to the number of living cells; to this purpose, CellTiter-Glo^TM^ cell viability assay kit (Promega), assaying ATP as a marker for basal metabolic activity, was used.

### 2.11. Statistical Analysis

Dose–response curves, box and whiskers plots, bar plots and related statistical analysis were obtained by using GraphPad Prism software (version 8.0.2, San Diego, CA, USA).

## 3. Results

### 3.1. Resveratrol Cytotoxicity

**Figure 1:**
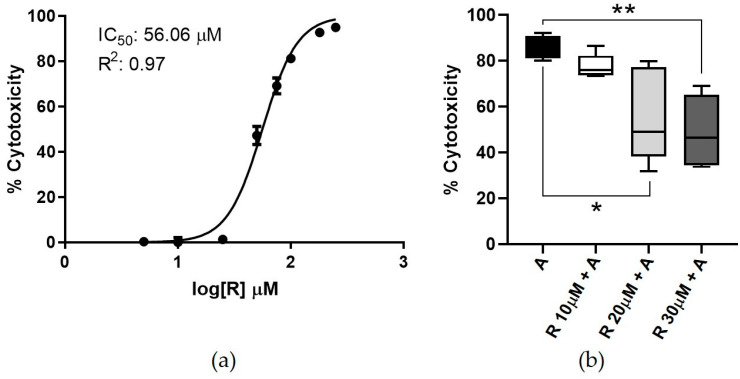
Cytotoxicity. (**a**) Resveratrol dose–response curve in BFH12 cells (64 h), based on three independent cell culture experiments, each one run in sextuplicate. Data are expressed in mean cytotoxicity rate ± standard error of the mean (SEM). IC_50_ and R^2^ are also reported. (**b**) AFB1 cytotoxicity in presence of R (16 + 48 h). The box and whiskers plots report the viability of BFH12 cells pre-treated with R increasing concentrations (10, 20, and 30 μM) and exposed for 48 h to a combination of AFB1 3.6 μM and R (same concentration reported above). ** *p* ≤ 0.01, * *p* ≤ 0.05 (Kruskal–Wallis and Dunn’s multicomparisons tests; the mean of each condition was compared with the mean of the AFB1 condition). Graphs were obtained by means of GraphPad prism software. R = resveratrol; A = AFB1.

Cells were exposed for 16 + 48 h to increasing R concentrations, and dose/response curves were built to define the corresponding IC_50_ values. After 64 h of incubation, the IC_50_ value was 56.06 μM (R^2^ = 0.97) (Figure 1a).

The cytotoxicity assessed following the AFB1 and R co-treatment showed that R mitigated AFB1-induced cytotoxicity in a dose-dependent manner. In particular, the highest R dose (i.e., 30 μM) significantly reduced the median AFB1 cytotoxicity, dropping from 86.00% to 49.22% (Figure 1b).

### 3.2. Aflatoxin B1 Biotransformation in BFH12 Cells

**Figure 2:**
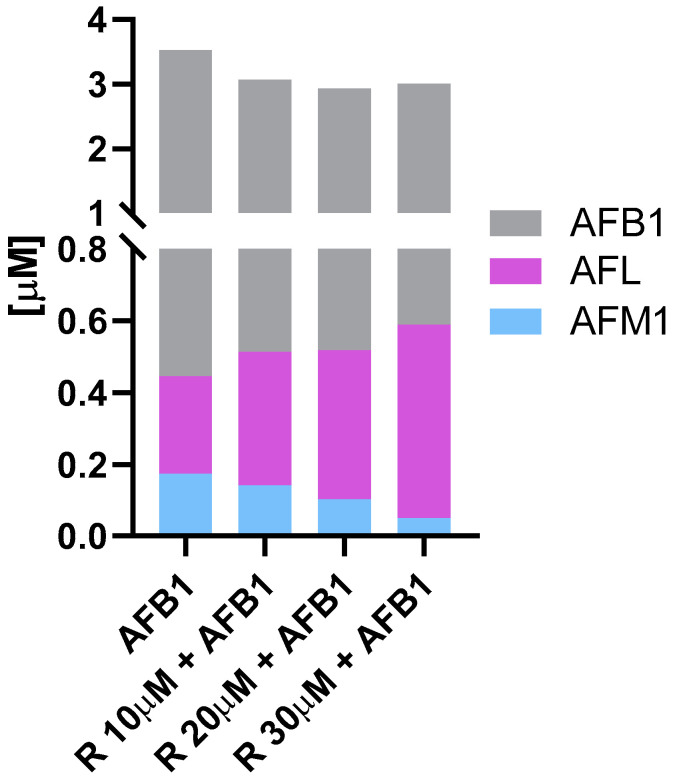
AFB1 biotransformation. The cumulative bar chart reports the micromolar (μM) amount of AFB1, AFM1, and AFL detected in the medium of BFH12 cells exposed to AFB1 alone or in combination with 10, 20, or 30 μM of R. R = resveratrol.3.3. Target gene expression analysis.

Bovine foetal hepatocytes metabolized AFB1, producing and releasing into the medium its foremost derivatives, i.e., AFM1 and AFL (Figure S2). Resveratrol significantly decreased the amount of AFM1 in a dose-dependent manner, reducing this metabolite from 57.00 ng/mL (i.e., 0.174 μM) to 16.25 ng/mL (i.e., 0.050 μM). On the contrary, R increased the production of AFL, which reached the highest amount (i.e., 169.50 ng/mL, or 0.539 μM) in the medium of cells co-treated with R 30 μM and AFB1. A comprehensive picture of biotransformation processes occurring in BFH12 exposed to AFB1, alone or in combination with R, is provided in Figure 2. Beside summarizing the impact of R on AFM1 and AFL formation, Figure 2 also highlights a decrease in AFB1 concentration in presence of R, yet the variations are above the threshold of significance.

In cellular pellets, AFM1 and AFL were not detected, while the amount of cellular AFB1 was generally low in all different experimental conditions (approximately 12 ng/mL, or 0.038 μM).

### 3.3. Target Gene Expression Analysis

Some key genes involved in phase I and II biotransformation, AHR pathways, and antioxidant mechanisms were affected by R alone or co-administered with AFB1 (Figure S3). The observed patterns of gene expression demonstrated that R effects are dose-dependent, and the highest dose (i.e., 30 μM) produced maximal variations. The mRNA levels of CYP1A1, CYP3A28 (the orthologue of human CYP3A4, as demonstrated in [68]), and GSTA1 were slightly induced by R. Moreover, in cells incubated with R and AFB1, CYP1A1 and the aryl hydrocarbon receptor repressor (AHRR) were significantly up- and downregulated, respectively.

To obtain net transcriptional results, the RNA-seq studies have been carried out only in cells treated with the highest R concentration.

### 3.4. Whole-Transcriptome Differential Expression Analysis

A total of 291,836,355 raw reads were sequenced and deposited in GeneBank under the BioProject accession PRJNA627332.

Raw reads of all samples passed quality control measures. After trimming and rRNAs removal, approximately 24 million reads per sample were retained. About 99% of the reads obtained from each RNA-seq library mapped to the *B. taurus* reference genome. In Table S1 we reported the number of raw reads passing the filters and the number of filtered reads mapping to the cow genome. The MDS plot provided in Figure S4 shows an unsupervised clustering of the samples. The first dimension (x axis) clearly separates cells treated with AFB1 (alone or in combination) from those which did not receive AFB1. Transcriptional profiles observed in control cells and those exposed to R appear very similar, thus forming a unique cluster in the plot. Biological variability within CTRL, AFB1, and R experimental groups is narrow as demonstrated by the coherent clusters formed by replicas. Finally, circles representing the whole transcriptional pattern of cells co-treated with R and AFB1 are more dispersed in the Cartesian plane, thus indicating a certain biological variability between replicas.

In cells incubated with R alone, a total of 5 DEGs were found; compared to controls, 4 genes were overexpressed, while only one gene was downregulated. The EdgeR output of the DE analysis conducted in this study was reported in Table S2. Four out of 5 DEGs were involved in inflammatory processes: fatty acid desaturase 1 (FADS1; logFC = 0.80), high mobility group AT-hook 1 (HMGA1; logFC = 1.01), secreted phosphoprotein 1 (SPP1; logFC = 2.03), and signal peptide, CUB domain and EGF-like domain containing 1 (SCUBE1; logFC = −3.20).

**Figure 3:**
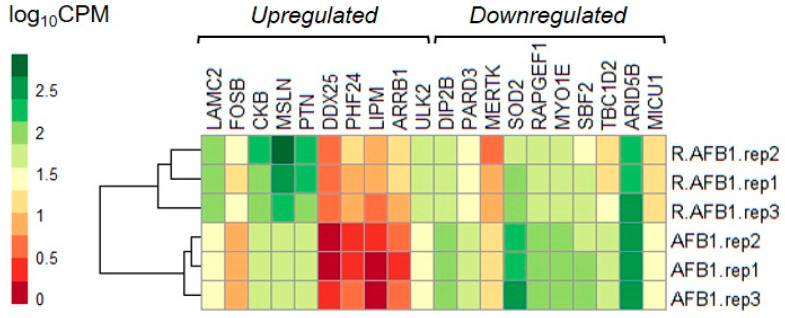
Heatmap of top-10 up- and downregulated genes by R+AFB1 co-treatment (vs. AFB1). The graph was constructed in R environment using the *pheatmap* package and using as input the normalized log_10_CPM (Counts Per Million). R = resveratrol; A = AFB1.

Gene expression profiles of cells co-treated with R and AFB1 were compared to those of cells treated with AFB1 alone. In BFH12 cells co-treated with R and AFB1, a total of 840 DEGs were identified. The majority of significant genes, i.e., 647, were upregulated, while 193 were downregulated. The EdgeR output of the DE analysis conducted in this study is reported in Table S2. The top-10 up- and downregulated genes are reported in a heatmap (Figure 3). Noteworthy, among the top-10 upregulated genes we found cancer-related genes such as FosB proto-oncogene (FOSB; logFC = 1.89), mesothelin (MSLN; logFC = 3.06), and arrestin beta 1 (ARRB1; logFC = 1.89). The most significant upregulated gene was laminin subunit gamma 2 (LAMC2; logFC = 1.69), a component of the ECM-receptor interaction pathway. Worth of note, an autophagy-activating kinase (ULK2; logFC = 0.87) was also found.

Among the top-10 downregulated genes, superoxide dismutase 2 (SOD2; logFC = −1.30) showed the highest fold change. A further gene involved in regulating oxidative stress and inhibited in R + AFB1 co-treated cells was mitochondrial calcium uptake 1 (MICU1; logFC = −0.97). Besides, genes with a role in apoptosis were also found, i.e., MER proto-oncogene, tyrosine kinase (MERTK; logFC = −1.30), Rap guanine nucleotide exchange factor 1 (RAPGEF1; logFC = −0.74).

**Figure 4:**
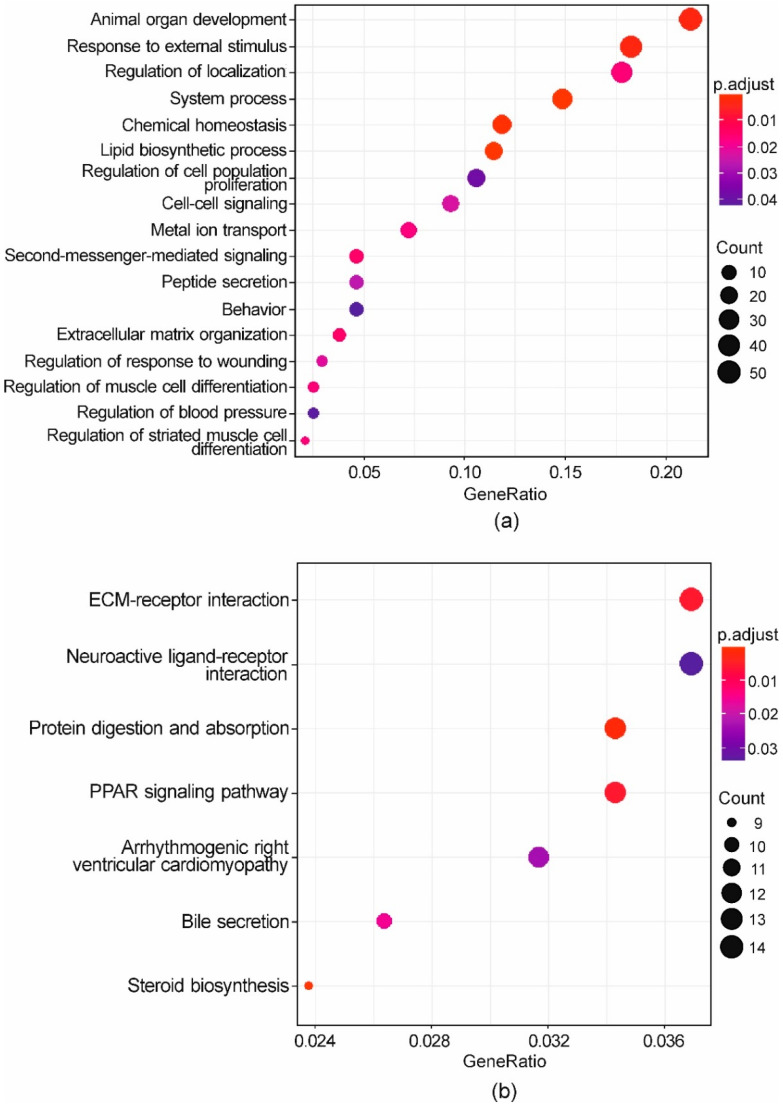
Functional enrichment of genes regulated by R+AFB1 co-treatment (vs. AFB1). Dot plots representing enriched BPs (**a**) and KEGG pathways (**b**). Dot size reflects the number of DEGs in each enriched term. The colour gradient reflects the significance level of each term. *p*-values were adjusted using the Benjamini-Hochberg method.

The enrichment analysis, carried out on the complete list of DEGs (both up- and downregulated), resulted in 17 significant Biological Processes (BPs) and 7 KEGG pathways (Figure 4, Table S3). Besides the very general terms “system process” (GO:0003008; 35 genes) and “chemical homeostasis” (GO:0048878; 28 genes), a significant BP was “lipid biosynthetic process” (GO:0008610; 27 genes). As regards this term, genes coding for key enzymes in cholesterol formation like 3-hydroxy-3-methylglutaryl-CoA reductase (HMGCR; logFC = 0.78), lanosterol synthase (LS; logFC = 1.44), and NAD(P) dependent steroid dehydrogenase-like (NSDHL; logFC = 1.07) were upregulated. Several DEGs representing this term play a role in inflammatory processes. This is the case of fatty acid binding protein 5 (FABP5; logFC = 1.14), apolipoprotein A1 (APOA1; logFC = 2.17), low density lipoprotein receptor (LDLR; logFC = 0.99), prostaglandin I2 synthase (PTGIS; logFC = 1.73), phospholipase A2 group IVA (PLA2G4A; logFC = −1.14), transmembrane 7 superfamily member 2 (TM7SF2; logFC = −1.00), and nuclear receptor subfamily 1 group D member 1 (NR1D1; logFC = −0.87). While R administered alone induced FADS1, the co-treatment with AFB1 upregulated the expression of both FADS1 (logFC = 0.74) and FADS2 (logFC = 1.33), an analogue to FADS1 but desaturating fatty acid chain at specific positions.

Notably, a significantly enriched BP was also the “response to external stimulus” (GO:0009605; 43 genes) with genes like aquaporin 1 (AQP1; logFC = 2.02), fos proto-oncogene, AP-1 transcription factor subunit (FOS; logFC = 1.06), nuclear receptor subfamily 1 group H member 4 (NR1H4; logFC = 3.32), serpin family G member 1 (SERPING1; logFC = 1.87), and fibronectin type III domain containing 4 (FNDC4; logFC = 1.27). All these genes are known to be involved in anti-inflammatory processes. Furthermore, the expression of some pro-inflammatory cytokines like C-X-C motif chemokine ligand 9 (CXCL9; logFC = 2.79), CXCL10 (logFC = 3.11), CXCL11 (logFC = 2.20), and CXCL16 (logFC = 0.64) was significantly induced. As for downregulated genes, some of them have a pro-carcinogenic activity, like the activated leukocyte cell adhesion molecule (ALCAM; logFC = −0.61), and semaphorin 3C (SEMA3C; logFC = −1.24). The OMA1 zinc metallopeptidase (OMA1; logFC = −0.97) is involved in apoptosis, and DNA damage inducible transcript 3 (DDIT3; logFC = −0.69) in cell stress and DNA damage.

Differentially expressed genes playing a role in processes involving cell–cell communication were particularly represented, resulting in the significant enrichment of the BP “extracellular matrix organization” (GO:0030198; 9 genes), “second-messenger-mediated signalling” (GO:0019932; 11 genes), and “cell-cell signalling” (GO:0007267; 22 genes).

As far as KEGG enrichment, the significance of “steroid biosynthesis” (bta00100; 9 genes) is consistent with the enriched BP “lipid biosynthetic process” (see above). Among significant KEGGs, “PPAR signalling pathway” (bta03320; 13 genes) appears particularly interesting.

**Figure 5:**
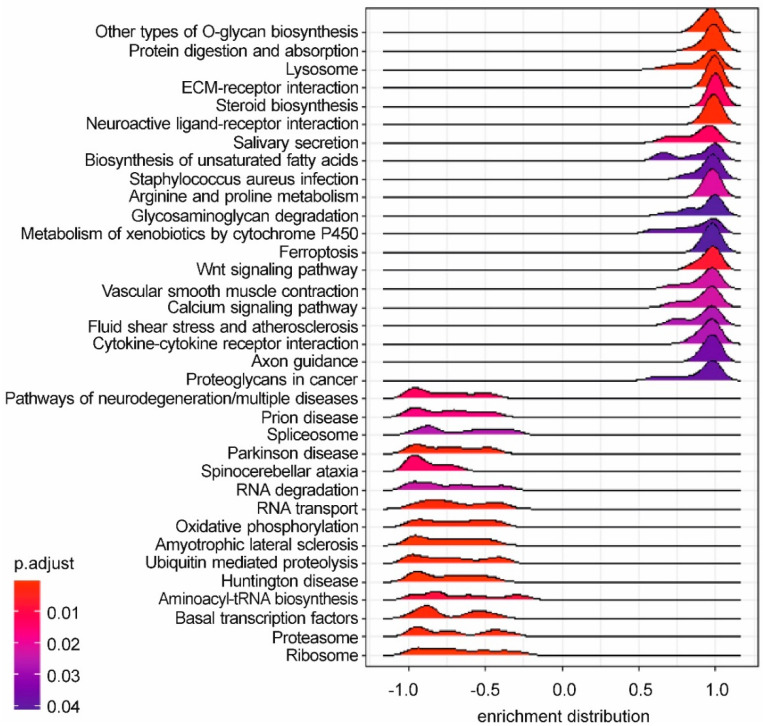
Gene Set Enrichment Analysis: R+AFB1 vs. AFB1. The ridgeplot visualizes the expression distributions of core enriched genes for GSEA enriched KEGG pathways. Gradient colour reflects the adjusted *p*-values (Benjamini–Hochberg method).

The GSEA conducted on the output of the DE analysis resulted in 35 significant KEGG pathways (Figure 5, Table S4). Among them, 20 were positively enriched (Normalized Enrichment Score > 0), meaning that a relevant number of genes belonging to these terms were upregulated in R+AFB1 compared to AFB1. The remaining 15 pathways were negatively enriched (Normalized Enrichment Score < 0), meaning that a relevant number of genes belonging to these terms were downregulated in R+AFB1 compared to AFB1. Firstly, some positively enriched KEGG pathways, like “ECM-receptor interaction” (bta04512; 58 genes), “steroid biosynthesis” (bta00100; 17 genes), “metabolism of xenobiotics by cytochrome P450” (bta00980; 31 genes), and “axon guidance” (bta04360; 151 genes) confirmed some of the results of the over-representation test (see below). Secondly, the positive enrichment of “glycosaminoglycan degradation” (bta00531; 17 genes) and “lysosome” (bta04142; 114 genes) demonstrated an increased activity of these organelles and their peculiar biochemical reactions, such as the catabolism of glycosaminoglycans (GAGs), structural constituents of the proteoglycans. Furthermore, in the present study the pathway “proteoglycans in cancer” (bta05205; 169 genes) was activated by R and AFB1 co-treatment. Fatty acids metabolism was also impacted by R, as demonstrated by the positive enrichment of “biosynthesis of unsaturated fatty acids” pathway (bta01040; 26 genes). Interestingly, the KEGG pathway “ferroptosis” (bta04216; 35 genes) was significantly activated. Ferroptosis is a type of regulated oxidative cell death that is induced by the accumulation of iron-mediated lipid peroxidation.

Among the downregulated gene sets, we found some terms related to RNA processing, like “spliceosome” (bta03040; 124 genes), “RNA degradation” (bta03018; 73 genes), “RNA transport” (bta03013, 150 genes), and “aminoacyl-tRNA biosynthesis” (bta00970; 43 genes). Processes related to protein synthesis and degradation were also suppressed, as demonstrated by the negatively enriched KEGG pathways like “ubiquitin mediated proteolysis” (bta04120; 133 genes), “proteasome” (bta03050; 43 genes), and “ribosome” (bta03010; 128 genes).

### 3.5. Oxidative Stress

**Figure 6:**
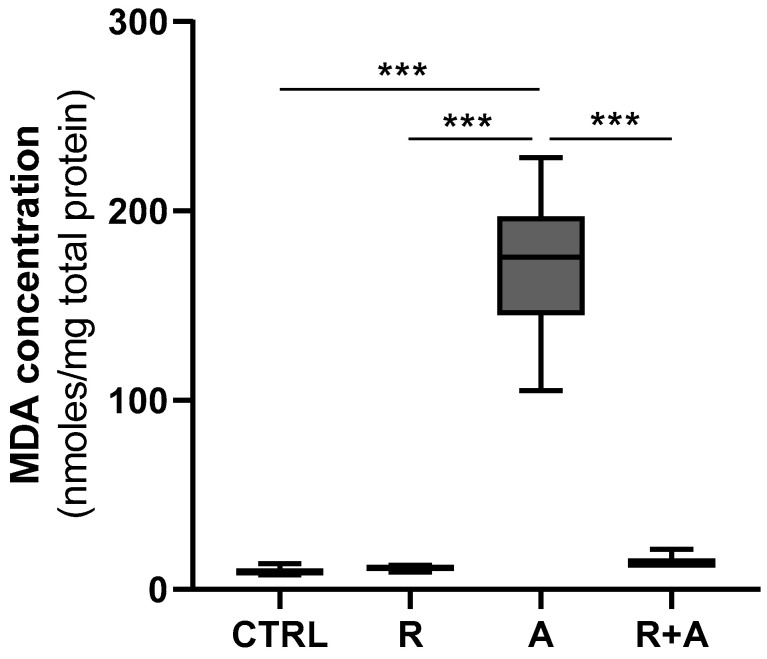
Lipid peroxidation. Box and whiskers plot reports the MDA content in the four different experimental conditions. The statistical comparisons were established between the median MDA concentration in cells exposed to AFB1 and the median MDA concentration observed in all other experimental conditions. Data are expressed as median concentration and data distribution (i.e., quartiles). For each experimental condition, six independent cell culture experiments were performed. CTRL condition corresponds to cells exposed to PCB126 only. R = resveratrol; A = AFB1. *** *p* < 0.001 (one-way ANOVA, followed by Dunnett’s multi-comparisons test).

In cells, oxidative stress may result in lipid peroxidation. To confirm the pro-oxidant effects of AFB1 and the potential benefits of R we measured the amount of MDA, which is a marker of lipid peroxidation. The amount of MDA significantly increased in BFH12 cells exposed to AFB1 compared to control conditions (Figure 6). The co-incubation with R significantly reduced the MDA content (Figure 6), thus demonstrating the potential of this natural extract in mitigating AFB1-induced oxidative damage.

### 3.6. Diaphorase Enzymatic Activity

The activity of NQO1 was selected as a marker of the cell antioxidant status and, additionally, to confirm the trend observed for this specific target at the mRNA level (qPCR and RNA-seq: Figure S3, Table S2). Moreover, this assay was chosen to allow a comparison with findings of our previous study in which we assessed the effects of another antioxidant in BFH12 cells, i.e., curcumin [21].

**Figure 7:**
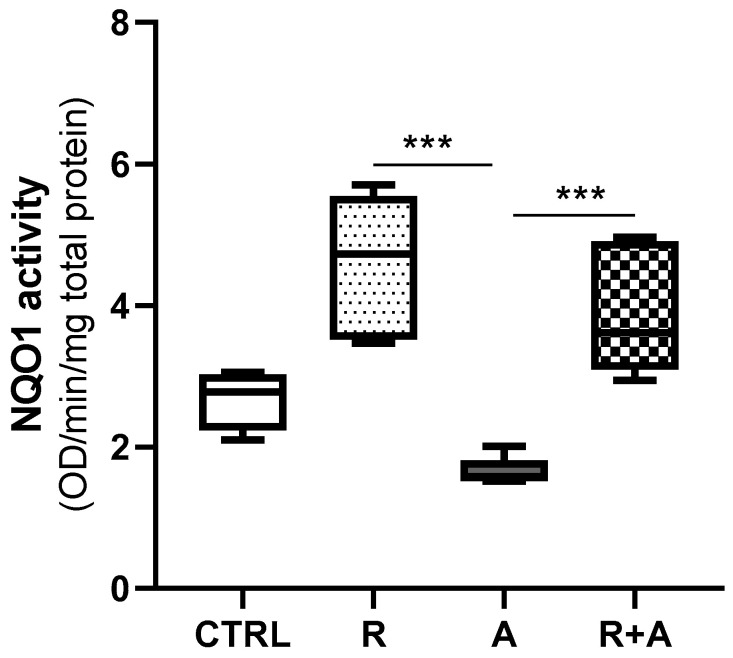
NQO1 enzyme activity. Box and whiskers plot reports the diaphorase activity in different experimental conditions. The statistical comparisons were established between the median NQO1 activity in cells exposed to AFB1 and the median NQO1 activity observed in the other experimental conditions. For each experimental condition, six independent cell culture experiments were performed. Data are expressed as median optical density (OD) observed per min per mg of total protein. CTRL condition corresponds to cells exposed to PCB126 only. R = resveratrol; A = AFB1. *** *p* < 0.001 (one-way ANOVA, followed by Dunnett’s multi-comparisons test).

Aflatoxin B1 reduced the antioxidant activity of NQO1 when compared to CTRL, (Figure 7), though the variation was close but not statistically significant (*p* = 0.067). However, R alone or in combination with AFB1 significantly induced the NQO1 enzymatic activity, bringing it above that of CTRL cells (Figure 7). This result further corroborates evidence of the antioxidant properties of R.

### 3.7. Cytochrome P4503A Catalytic Activity

**Figure 8:**
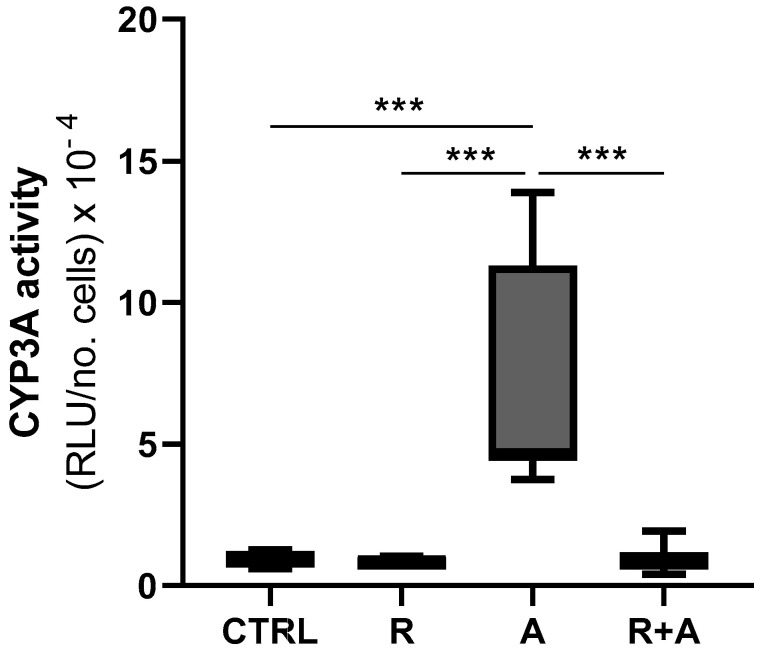
CYP3A enzyme activity. Box and whiskers plot reports the CYP3A activity in different experimental conditions. The statistical comparisons were established between the median CYP3A activity in cells exposed to AFB1 and the CYP3A activity observed in the other experimental conditions. For each experimental condition, seven independent cell culture experiments were performed. Data are expressed as relative luminescence units (RLU) normalized to the total number of alive cells. CTRL condition corresponds to cells exposed to PCB126 only. R = resveratrol; A = AFB1. *** *p* < 0.001 (one-way ANOVA, followed by Dunnett’s multi-comparisons test).

Cells exposed to AFB1 showed a statistically significant increase in CYP3A activity compared to basal conditions (*p* < 0.001, Figure 8). Conversely, the combined exposure to AFB1 and R considerably reduced the CYP3A activity, bringing it back to CTRL levels (Figure 8).

To further ascertain the impact of R on the activity of this CYP enzyme, playing a crucial role in AFB1 metabolism, we observed how R alone reduces CYP3A catalytic activity in a dose-dependent way, being 30 μM the first significant concentration (Figure S5).

## 4. Discussion

To date, the protective role of R against AFB1 in farm animals has been scarcely studied, and the molecular mechanisms underlying the protective role of this natural polyphenol has never been investigated in depth. A couple of studies have been conducted to elucidate the effects of R in bovine mammary cells exposed to AFB1 [26,31]. As for the liver, a primary target of AFs toxicity, an in vivo study has been performed in broilers fed with R in combination with AFB1 [32]. Overall, whole transcriptomic changes resulting from co-treatment with R and AFs have never been elucidated, not even in mammals.

### 4.1. Resveratrol Cytotoxicity

The IC_50_ of R at 64 h was similar to that reported in bovine mammary cells (BME) experimentally exposed for 48 h (i.e., approximately 50 μM) [26]. This result is consistent with the IC_50_ observed at 48 h in six human cancer cell lines, ranging from 70 to 150 μM [69]. Resveratrol cytotoxicity has been recently investigated also in normal cell lines such as the renal tubular epithelial cell line HK-2 and human liver cell line L02, exhibiting an IC_50_ at 72 h of approximately 150 and 200 μM, respectively [70]. A direct comparison between R IC_50_ in normal human and bovine hepatocytes cannot be established, because BFH12 are foetal-derived cells; moreover, to our knowledge, no study has yet investigated the cytotoxicity of this natural compound in primary bovine hepatocytes. However, the cytotoxicity data here obtained let us suggest that bovine hepatocytes are more sensitive to R than the human counterpart. Worth of note, when comparing IC_50_ of different natural antioxidant extracts at 64 h in BFH12, R shows a cytotoxicity potential similar to that observed for quercetin (Pauletto et al., manuscript in preparation), but considerably lower than those reported for curcuminoids [21].

The significant decrease in AFB1-induced cytotoxicity was crucial in demonstrating the positive impact of R in cattle, and confirmed the extraordinary potential of this natural extract. In BFH12 exposed to AFB1, curcuminoids were already proven to increase cell viability [21], but such an effect was lower compared to that observed in the present study. In agreement with this study, in BME-UV1 cells 5 μM R provided some protection against the cytotoxicity observed after 48 h of AFB1 exposure (96 nM), increasing cell viability from 40% up to 60% [26]. Moreover, in agreement with data we obtained in BFH12, the study by Ghadiri et al. showed a greater ability of R, compared to curcumin, in mitigating AFB1-induced cellular death. Such an evidence let us hypothesize that the potential benefits of R in cattle might be even stronger than curcumin. Considering the higher MDA levels observed in AFB1-treated compared to R and AFB1 co-treated cells, we hypothesize that oxidative stress concurs to increase cell damage and consequently the risk of cell death.

### 4.2. Aflatoxin B1 Biotransformation

The amount of AFM1, the most toxic AFB1 derivative [71], appeared inversely correlated with R concentrations, as previously observed in BFH12 cells co-treated with curcuminoids and AFB1 [21]. As hypothesized for curcuminoids, it is likely that R reduces AFM1 production by targeting the major enzymes involved in AFM1 hepatic formation [72], and most probably CYP3A. Specifically, CYP3A catalytic activity was enhanced by AFB1 alone, while it was significantly reduced by 30 and 40 µM R. Likewise, R in combination with AFB1 was shown to reverse the mycotoxin-dependent increase in CYP3A enzymatic activity. Overall, these results would suggest CYP3A as the main CYP involved in AFM1 synthesis in BFH12 cells. However, more specific biomolecular studies are needed to confirm such a hypothesis.

Conversely, the amount of highly toxic AFL increased with R concentration, thus indicating that this natural polyphenol impacts on AFB1 reduction, the reaction forming AFL in the liver [73]. Aflatoxicol can be reconverted to AFB1, thereby serving as a reservoir of AFB1 and thus prolonging the AFB1 residency time in the liver [74]. Despite that, a very recent study demonstrated in poultry species that high rate of AFL production is associated with minor sensitivity to AFB1 [75]. Indeed, the authors postulated that an efficient AFB1 biotransformation to AFL reduces the amount of AFB1 available for bioactivation, thus mitigating the toxicity of AFBO.

Overall, the impact of R on AFM1 and AFL formation seems to be consistent with a beneficial influence of this natural polyphenol.

### 4.3. Resveratrol Transcriptional Effects

Overall, R administered alone induced neglectable transcriptional changes. Worth of mention, in this same cellular model, curcuminoids had a considerable impact on gene transcription, as they significantly affected the expression of hundreds of genes [21]. Notably, curcuminoids were less effective in reducing AFB1-induced toxicity; hence, it is likely that polyphenols that highly perturb BFH12 transcriptional profiles might not be the best choice to improve cell viability.

The most significant DEG was a gene involved in lipid metabolism, the desaturase FADS1, whose transcription was significantly induced by R. This result is consistent with previous literature reporting FADS1 induction in human HepG2 treated with 40 μM R [76]. FADS1 plays a role in the synthesis of long chain polyunsaturated fatty acids (PUFA) occurring in liver, and controls the metabolism of inflammatory lipids like prostaglandin E2, critical for efficient acute inflammatory response and maintenance of epithelium homeostasis. Notably, in human melanocytes, FADS1 downregulation induced cell cycle arrest and cell death via ROS generation and mitochondria-mediated apoptosis [77]. Indirectly, this evidence let us think that FADS1 induction in BFH12 treated with R might have beneficial effects. Notably, FADS1 induction possibly occurs via a PPARα activation mediated by R, as previously postulated [52,76,78].

Two key players in inflammatory processes, cell proliferation and survival by regulating apoptosis, i.e., HMGA1 and SPP1 (also known as osteopontin) [79,80,81], were also induced by R. The former is considered as an oncogene, and its overexpression is a common feature of several human malignancies [82]. Interestingly, an association between the expression of SIRT1, known to be induced by R, and HMGA1 was highlighted in lung cancer [83]. It is not known how the transcriptional increase in this oncogene triggered by R might affect cell functioning, although its association with SIRT1 let us think that it exerts beneficial effects. Intriguingly, it has been hypothesized in rat hepatic stellate cell line (HSC) that the upregulation of HMGA1 occurring after curcumin exposure activates cells senescence, thus preventing fibrogenic response to liver tissue damage [84]. Similarly, we suggest that R has the same ability in BFH12, and senescence mediated by HMGA1 (together with P53 and PPARγ in rat HSCs) might reduce cell injury. Osteopontin is a crucial player in wound healing processes as it serves as a chemotactic molecule to promote the migration of inflammatory cells to the wound site, where it acts as an adhesive protein to retain cells. For instance, osteopontin has been proposed to be a bone repair mediator, and its transcription was induced in skulls collected from rats treated with 10 mg/kg R for 30 days [85]. Likewise, immunohistochemical analyses revealed that R induces osteopontin in rats, and improved alveolar socket healing [86].

The sole gene downregulated by R, SCUBE1, a cell surface glycoprotein found in platelet and endothelial cells [87], was particularly interesting. A very recent study [88] has demonstrated that SCUBE1 is an independent prognostic factor in septic patients, and positive correlates with accepted inflammatory biomarkers and “Acute Physiology And Chronic Health Evaluation II” (APACHE-2), a severity-of-disease classification system [89]. Moreover, accumulation of this protein in blood is associated with vascular dysfunctions like atherosclerotic lesions or ischemic stroke [90], and ovarian impairment [91,92]. Overall, considering the above reported functions of SCUBE1, we suggest that its great downregulation in BFH12 exposed to R (nearly ten times less expressed compared to control) is indicative of very low activation of inflammatory processes.

### 4.4. Transcriptional Changes Underlying R Potential as an Anti-AFB1

If R alone did not deeply interfere with BFH12 transcriptional patterns, the co-treatment with AFB1 greatly modulated the whole transcriptome when compared to that of cells exposed to AFB1 only. This let us think that the potential of this natural polyphenol is maximal in presence of a stressful event, like AFB1. Indeed, R pre-treatment was effective in counteracting molecular processes triggered by AFB1, and promoting cell survival.

The functional analysis pointed out some interesting pathways underlying the mechanisms by which R is able to mitigate AFB1-induced toxicity. Among the most significantly enriched BP, we found “lipid biosynthetic process”. This term is mainly associated with the tight connection between cholesterol metabolism and inflammation, and is mostly represented by genes whose upregulation aims at decreasing AFB1-induced inflammatory events. Among these ones we found APOA1, PTGIS, and LDLR. The former codes for the major protein component of high-density lipoprotein (HDL), widely known for regulating cholesterol trafficking. It exhibits tumor-suppressive activity by promoting anti-inflammatory processes and modulating immune responses [93,94]. The second transcript (PTGIS) catalyzes the biosynthesis and metabolism of eicosanoids; its overexpression was reported to prevent the development of murine lung tumors [95], inhibit the activation of HSCs and alleviate liver fibrosis in mice [96]. Finally, LDLR mediates binding and endocytosis of low-density lipoproteins (LDLs), and it has been previously found to be upregulated by R in cultured hepatocytes [97,98]. As oxidized LDLs is a marker of inflammation, the overexpression of this receptor, eliminating LDLs, might be considered as a beneficial and anti-inflammatory molecular event in BFH12 co-treated with R and AFB1.

Two out of the 5 downregulated genes representing the lipid biosynthesis term (i.e., PLA2G4A and TM7SF2) were most likely implicated in mitigating the inflammatory response, thus reducing cell injury. The former gene (PLA2G4A) encodes for an enzyme that catalyzes the hydrolysis of membrane phospholipids to release arachidonic acid, a hinge molecule in eicosanoids synthetic pathway. Eicosanoids are lipid-based cellular hormones key for the inflammatory response. Notably, in rat mesangial cells pre-treated with R derivatives and stimulated with the pro-inflammatory cytokine TNF-α, Lee et al. reported an inhibition of PLA2G4A protein expression, thus suggesting an anti-inflammatory activity of these natural compounds [99]. Likewise, in our previous study, we observed a transcriptional decrease in PLA2G4A in BFH12 pre-treated with curcumin and exposed to AFB1 [21]. A dual activity has been proposed for TM7SF2; besides actively participating in cholesterol biosynthesis, it might be linked to the cellular response to endoplasmic reticulum stressors [100]. Its downregulation might be indicative of an anti-inflammatory response triggered by R pre-treatment. A similar hypothesis has been drawn by Gatticchi et al., who observed in TM7SF2 knockout mice that the gene deficiency is responsible in kidney for the inhibition of the signalling mediated by the nuclear factor kappa-light-chain-enhancer of activated B cells (NFkB), thus dampening the inflammatory response induced by LPS and leading to a reduced renal damage [101].

The enriched BP “response to external stimulus” was represented by some key genes which provide additional information about the molecular mechanisms underlying the R protective role against AFB1-induced toxicity in BFH12. For instance, AQP1 (upregulated by R) might exert a cytoprotective role and help to alleviate the inflammatory reaction induced by AFB1. Its overexpression has been previously suggested to confer survival advantage in LPS-induced acute kidney injury, both in vivo in rats [102]) and in vitro in human proximal tubule cell line [103]. Similarly, FOS, a key regulator in inflammation, apoptosis and the immune system, was induced by R. Interestingly, in macrophages FOS suppresses the expression of inducible nitric oxide synthase and pro-inflammatory cytokines (e.g., TNF-α), and increase the expression of anti-inflammatory genes, such as suppressor of cytokine signalling 3 (SOCS3) [104], whose mRNA was upregulated by R also in our cell model. Notably, in human embryonic kidney 293 cells, CACO-2 cells, and murine C17.2 neural stem cells, the transcription of FOS protooncogene was reported to be stimulated by 20 μM R [105]. The transcription receptor NR1H4 (also known as Farnesoid X Receptor, FXR), regulating the inflammatory response and greatly expressed in liver, was largely overexpressed in AFB1-exposed cells pre-treated with R. The significance upregulation of FXR in the present study might be related to the anti-inflammatory activity of this transcription factor. In fact, FXR has been previously shown to confer protection in several animal models of innate intestinal and hepatic inflammation [106]. A similar role is most likely exerted by SERPING1 (also known as C1INH) and FNDC4, both upregulated by R in BFH12. The former is a complement inhibitor that has demonstrated the ability to effectively reduce complement activation, thus dampening tissue inflammatory reactions, both in vivo and in vitro in pig liver [107]. The latter codes for a secreted protein recently proven to act as an anti-inflammatory factor in mouse intestine and colon [108]. In our study, FNDC4 was downregulated in BFH12 cells exposed to AFB1 [63], but a pre-treatment with R enhanced its transcription, that most likely improved cell survival, and downregulated the expression of pro-inflammatory genes. Accordingly, we found the same pattern of expression in BFH12 pre-treated with curcumin [21].

In the presence of R, CXCR3 ligands (CXCL9, CXCL10, and CXCL11), that are pro-inflammatory cytokines, were upregulated. Interestingly, CXCL10 (the IFN-inducible protein-10/IP-10) is a general marker of hepatic inflammation and injury, but it has also shown hepatoprotective effects in mice, as it plays a role in hepatic repair and regeneration [109,110]. However, this result is in contrast with other studies reporting that the transcriptional inhibition of these genes, mediated by natural extracts, might have a protective role in liver. For instance, in mice a decrease in CXCL10 production has been suggested to partly account for the inhibitory effect of curcumin on CD4+ T cells infiltration in the liver [111]. Furthermore, in human retinal pigment epithelium cells treated with pro-inflammatory cytokines, a pre-treatment with 50 μM R inhibited the expression of CXCL9 and CXCL11 [112]. In vivo, a higher CXCL9 and CXCL10 expression was associated with animals with the greatest degree of liver injury [113,114]. Nonetheless, it should be noted that the expression of these cytokines might change over time during liver injury, and these changes are crucial in determining the fate of cells, as hypothesized in vivo in human serum [115]. Notably, in a previous study we demonstrated that in BFH12 exposed to AFB1 the expression of CXCR3 ligands was largely downregulated [63]. Here, we show that R reverts this trend of expression, and this let us think that CXCL9, CXCL10, and CXCL11 induction by R somehow counteracts AFB1 toxic effects. More research is needed to shed light in the role of these important pro-inflammatory cytokines in mediating R anti-AFB1 activity in cattle.

Some interesting genes involved in cancer development and invasion, and belonging to the BP “response to external stimulus” were downregulated by R, thus suggesting that R might attenuate the activation of biological processes normally triggered by AFB1 stimulus and responsible of the hepatocarcinogenicity of this mycotoxin. This is the case of ALCAM (also known as CD166), SEMA3C, and OMA1. The first gene is a cell surface member of the immunoglobulin superfamily playing a pro-carcinogenic role in liver cancer [116]; interestingly, it has been reported to be inhibited in vitro by dietary polyphenols like curcumin [117]. The metastasis marker SEMA3C [118] is correlated with chronic hepatitis and significant fibrosis in human liver, too [119]. To our knowledge, ALCAM and SEMA3C have not been reported to be associated with mycotoxins toxicity. However, as in human liver cells an increase in their expression is involved in hepatic diseases, we might hypothesize that their downregulation by R is a beneficial effect that protects cells from AFB1-induced damage. Finally, OMA1, that was significantly downregulated by R, might be crucial in mitigating AFB1-induced toxicity, since it is a major mitochondrial factor for sensing and responding to cellular stress [120], including oxidative stress [121]. Moreover, OMA1 is responsible of optic atrophy-1 (OPA1) cleavage and mitochondrial fragmentation induced by H_2_O_2_, and mammalian cells lacking OMA1 showed protection against this mitochondrial stress [121]. In agreement with the present results, R reversed the increased mRNA levels of OMA1 observed in rat retina 7 days after ischemia/reperfusion injury [122]. Again, OMA1 has never been linked to AFB1. The evidence that lowering, or completely abolishing, the transcription of this gene provides protection from different oxidative stress in diverse mammalian cells, let us suggest that OMA1 downregulation triggered by R in BFH12 cells concurs to mitigate the detrimental effects of AFB1.

Careful analysis of the list of up- and downregulated genes pointed out some additional interesting mRNA changes triggered by R that most likely contribute to prevent cell damage and death occurring in cells exposed to AFB1.

For instance, CYP1B1 and CYP1A1 were both more expressed in cells co-treated with R and AFB1 compared to those treated with AFB1 only. Notably, if compared to control cells, the expression of these genes was significantly re-pressed by AFB1 [63]. Interestingly, both curcumin [21] and R showed the ability to revert the pattern of expression of CYP1A1 in BFH12. This let us suggest that an increased expression of this gene is a positive signature. Yet, it is possible that the activity of this enzyme is further regulated at a post-transcriptional level (e.g., protein translation, catalytic activity). Despite this, our previous investigations in BFH12 [21,63], and the changes triggered by R on CYP3A mRNA (not significant) and catalytic activity (significantly decreased) led us to hypothesize that CYP3A4, rather than CYP1A1, has a prominent role in AFB1 biotransformation. Moreover, if curcumin impacted both CYP3A expression and activity, R seems to target the activity of this enzyme, but not its transcription. This is in agreement with a previous study suggesting that R is a mechanism-based inactivator of human CYP3A4 [123]. The cytochrome P450 26B1 was the unique CYP downregulated by R, but it is not considered a classic xenobiotic metabolizing enzyme. Intriguingly, the expression of this gene was largely reduced also in BFH12 co-treated with curcumin [21], thus paving the idea that CYP26B1 might play an important role in BFH12 response to AFB1.

BFH12 pre-treated with R and then exposed to AFB1 exhibited transcriptional changes in some genes implicated in the antioxidant response and detoxification. Transferrin (TF), glutathione peroxidase 7 (GPX7), and peroxidasin (PXDN) were all significantly upregulated by R, while SOD2 was downregulated. Transferrin sequestrates iron, that produces free radicals, in a redox-inactive form, thus possessing an antioxidant capacity and providing a cytoprotective effect in various animal models [124]. Glutathione peroxidase hampers the accumulation of ROS in cells, and it participates in the correct protein folding as an intracellular stress sensor/transmitter to transfer the signal to its interacting proteins [125]. Similar to GPX7, GPX1 counteracts oxidative stress, but its transcription was inhibited by R, yet with marginal significance (FDR = 0.07). This result is in opposition with most of the published literature, highlighting a protective role of GPX1 induction under stressful conditions; but it is consistent with what we have observed in BFH12 co-treated with curcumin and AFB1 [21], suggesting shared molecular mechanism between these different natural polyphenols. Peroxidasin facilitates peroxidative reactions as it generates hypohalous acids by catalysing H_2_O_2_, and it is a target of NRF2 [126]. Notably, PXDN scavenges H_2_O_2_ in cardiovascular tissue, and it prevents oxidative stress in prostate cancer in vitro [127]. The downregulation of SOD2 is in contrast with most of studies assessing R transcriptional effects and reporting a significant induction of this mitochondrial enzyme [128,129,130]. However, this result is consistent with the effects induced by curcumin in the same cell model exposed to AFB1 [21], thus suggesting a peculiar transcriptional regulation for this crucial enzyme in bovine liver. Conversely, SOD1 was not significantly modulated by R. Notably, in the present study we focused on transcriptional modifications, but post-transcriptional regulations are likely to occur and affect the cells antioxidant capacity mediated by these enzymes.

Genes involved in detoxification were also significantly impacted by R. For instance, the microsomal glutathione S-transferase 1 (MGST1) was significantly induced by R and AFB1 co-treatment. This gene has a well-established role in the conjugation of electrophiles and oxidative stress protection [131]. Worth of note, its transcription was lower in BFH12 treated with AFB1 than in control cells [63], and, likewise to cells pre-treated with R, curcumin pre-treatment prevented AFB1-induced downregulation of this gene [21]. Nonetheless, in swine the placental mRNA expression of MGST1 was increased by dietary R [132]. Besides MGST1, glutathione S-transferase A2 (GSTA2) showed the same transcriptional behavior, but the corrected *p*-value was minimally below the threshold of significance (FDR = 0.056). This gene targets the same compounds of MGST1, including carcinogens, environmental toxins, and products of oxidative stress, by conjugation with glutathione. Overall, their transcriptional induction by R contribute to AFB1 detoxification and antioxidant mechanisms of response. Interestingly, in human and mice MGST1 and GSTA2 gene expression is regulated by NRF2 [133,134,135], a master regulator of the antioxidant response, whose expression was not significantly regulated in the present experiment. Based on our experience with this cell line, this is not particularly unexpected, since a similar transcriptional pattern was observed following the exposure to curcumin [21]. Conversely, the gene expression of NRF2 downstream genes (such as GSTs) appeared to be significantly affected. Therefore, it is likely that, in BFH12 cells, the polyphenols we tested might target NRF2 by affecting its protein synthesis and/or activity, rather than its level of transcription. Notably, some additional results let us suggest that R exerts it anti-AFB1 activity by regulating post transcriptionally some key genes. Pivotal examples could be NQO1 and CYP3A. Resveratrol greatly impacted their enzymatic activity, providing a protective mechanism against AFB1, but it did not affect their transcription at all.

As for the ABC transporters, ABCA1, multidrug-resistant protein 1 (MDR1), and ABCG2, showed a transcriptional pattern similar to that observed after curcumin pre-treatment: ABCA1 and MDR1 were downregulated, while ABCG2 was upregulated by R [21]. The downregulation of ABCA1, a transporter involved in the cholesterol efflux from cells and primarily responsible for the initiation of HDL formation, is in opposition to what reported in the literature. In fact, in human cell models, R was reported to promote the expression of cholesterol efflux proteins like ABCA1 [136]. Conversely, the downregulation of MDR1 by R, both at mRNA and protein level, has already been demonstrated in the context of drug-resistant cancer cells [137]. The present data do not allow us to clarify the role of MDR1 in cattle liver; however, it seems unlikely for this gene playing a prominent role in the AFB1 detoxification process [138]. Aflatoxins are ligands for ABCG2 and they have been demonstrated to affect the expression of this transporter [139]. A recent study conducted in bovine mammary epithelial cells (BMEs) has also demonstrated that ABCG2 (also known as breast cancer resistance protein, BCRP) is able to transport AFM1 [140]. The detailed mechanism of the aflatoxins transport by ABCG2 is still uncertain in cattle; however, ABCG2 upregulation might mitigate AFB1 toxicity decreasing its cellular uptake, as previously demonstrated in MDCK-II cells transduced with ABCG2 [139].

## 5. Conclusions

To the best of our knowledge, this is the first study assessing the potential benefits of R in AFB1-exposed bovine cells through an integrated approach encompassing cytotoxicity, whole-transcriptomics, and specific post-transcriptional confirmatory assays. Based on the present results, and the experiments that we have previously performed on this cell model, the following conclusions can be drawn. (1) Resveratrol pre-treatment hampered AFB1-induced cytotoxicity. (2) Resveratrol treatment scarcely affected the BFH12 transcriptome. (3) Resveratrol pre-treatment deeply modified the transcriptome of BFH12 cells exposed to AFB1. (4) The pathways mostly affected by R pre-treatment, and driving protective mechanisms against AFB1 toxicity, are related to lipid biosynthetic processes, inflammation, drug metabolism, and drug transport. (5) Prospective molecular studies are recommended to investigate the role played by specific pathways/genes in AFB1 mechanistic toxicology and, consequently, better characterize the protective role of R. (6) In vivo studies implementing R-supplemented diets are envisaged to assess R’s bioavailability and its beneficial effects on aflatoxicosis. (7) Compared to curcuminoids, R showed a greater potential to mitigate AFB1-induced toxicity; this suggests that R might be a better choice for further in vivo investigations assessing the introduction of this polyphenol in dairy cattle feeding strategies. Indeed, we expect that the beneficial effects, and the underneath molecular mechanisms, might occur also in vivo, yet with slight differences. Thus, in perspective, R-supplemented diets might improve health status of cattle exposed to AFB1-contaminated feed.

## Data Availability

Raw Illumina sequencing data have been deposited in GenBank (SRA) under the BioProject accession PRJNA627332.

## Supplementary Materials

The following are available online at https://www.mdpi.com/article/10.3390/antiox10081225/s1, Figure S1: Chemicals, Figure S2: Biotransformation of AFB1 in BFH12 cells, Figure S3. Real-time PCR: transcriptional changes induced by R, Figure S4. MDS plot, Figure S5. Dose dependent effects of resveratrol on CYP3A activity, Table S1. Sequencing and mapping results, Table S2. Differential expression analysis, Table S3. Over-representation analysis, Table S4. Gene Set Enrichment Analysis (KEGG pathways), File S1. R code.
