# Supplementary material for: Discovering the Protective Effects of Resveratrol on Aflatoxin B1-Induced Toxicity: A Whole Transcriptomic Study in a Bovine Hepatocyte Cell Line
Source: Antioxidants (Basel). 2021 Jul 29;10(8):1225. doi: 10.3390/antiox10081225 (PMC8388899; doi:10.3390/antiox10081225)
Supplement: Supplementary file 1 [file antioxidants-10-01225-s001.zip › antioxidants-1308402-supplementary/Supplementary_rev/Captions_rev.pdf]

Supplementary figures

Figure S1. Chemicals. Chemical structure of all the molecules considered in the present study, i.e. resveratrol, aflatoxins B1, M1, and aflatoxicol.

Figure S2. Biotransformation of AFB1 in BFH12 cells. Concentrations (µM) of AFM1 (a), and AFL (b) measured in the medium after 48 h of exposure to 3.6 µM AFB1 alone or in combination with R increasing concentrations (*i.e.*, at the end of the experiment, T96 h). Data are expressed as mean concentration ± standard deviation of five independent cell culture experiments. Different letters above error bars indicate significant differences ($p \leq 0.05$) among groups (Tukey's post-hoc test). R=resveratrol; A=AFB1.

Figure S3. Real-time PCR: transcriptional changes induced by R. The one-way ANOVA, followed by a Dunnett's multi-comparisons test, was carried out to compare the gene expression level of cells exposed to increasing doses of R (the lowest dose was used as control in the comparisons); significant variations were identified by black asterisks. The same statistical approach was adopted to investigate the effects of R treatment, separately: all the co-treatment conditions were compared to the AFB1 condition; significant variations were identified by red asterisks Data are expressed in fold change vs CTRL condition (*i.e.*, PCB126) ± standard deviation. CTRL condition, to which an arbitrary value of 1 was assigned, is not reported into the graph. R=resveratrol; A=AFB1. DMEs= drug metabolism enzymes; *$p \leq 0.05$; **$p \leq 0.01$.

Figure S4. MDS plot. The plot shows distances between expression profiles of the twelve RNA-seq libraries evaluated in this study. Biological replicates are represented by rep1, rep2, and rep3 suffix. RES=resveratrol; AF=AFB1.

Figure S5. Dose dependent effects of resveratrol on CYP3A activity. Bar plot reporting the CYP3A activity after increasing concentration of R or DMSO 0.1% (64 h). Data are obtained from two independent cell culture experiments and expressed as relative luminescence units (RLU) normalized to the total number of alive cells. R=resveratrol; A=AFB1. *$p \leq 0.05$; **$p \leq 0.01$ (one-way ANOVA, followed by Dunnett's multi-comparisons test).

Supplementary tables

Table S1. Sequencing and mapping results. The table reports the RNA-seq libraries sequenced, including for each of them: i) SRA accessions; ii) the number of raw reads obtained; iii) the number of reads after trimming and rRNAs removal; iv) the number of mapped reads (and the percentage of mapped reads).

Table S2. Differential expression analysis. The table reports the EdgeR *glmQLFTest* output for the tests performed ("R vs CTRL"; "R+AFB1 vs AFB1"). Ensembl gene description, gene name, log2 fold change (logFC), mean log count per millions (logCPM), nominal p-value and false discovery rate (FDR) are reported for all the genes evaluated. Genes significantly ($FDR \leq 0.05$; $log_2FC \geq 0.6$) upregulated by R pretreatment are highlighted in green colour, while genes downregulated by R are highlighted in red.

Table S3. Over-representation analysis. Table reports the over-represented BP terms and KEGG pathways in the list of DEGs resulted from the pair-wise comparison R+AFB1 vs AFB1. For each BP term (or KEGG pathway): gene ratio corresponds to number of annotated DEGs/number of DEGs possessing a GO/KEGG annotation, bg ratio represents the ratio between the number of annotated genes and the number of genes having a GO/KEGG annotation in the background list. P-values were adjusted by using the Benjamini-Hochberg (BH) procedure. *FDR: False Discovery Rate; GO: Gene Ontology; BP: Biological Processes; KEGG: Kyoto Encyclopedia of Genes and Genomes.*

Table S4. Gene Set Enrichment Analysis (KEGG pathways). Table report the over-represented KEGG terms in the comparison R+AFB1 vs AFB1. *NES: Normalized Enriched Score; BH: Benjamini-Hochberg; FDR: False Discovery Rate.*

Supplementary files

File S1. R code. Complete R code used for the differential gene expression analysis (edgeR), the data visualization, and the enrichment analysis.
