# Supplementary material for: Discovering the Protective Effects of Resveratrol on Aflatoxin B1-Induced Toxicity: A Whole Transcriptomic Study in a Bovine Hepatocyte Cell Line
Source: Antioxidants (Basel). 2021 Jul 29;10(8):1225. doi: 10.3390/antiox10081225 (PMC8388899; doi:10.3390/antiox10081225)
Supplement: Supplementary file 1 [file antioxidants-10-01225-s001.zip › antioxidants-1308402-supplementary/Supplementary_rev/FileS1.pdf]

File S1. R code. Complete R code used for the differential gene expression analysis (edgeR), the data visualization, and the enrichment analysis.

➔ *DIFFERENTIAL EXPRESSION ANALYSIS (EdgeR)*

Read data and set groups

```
>x<-read.delim("input.txt",row.names="Gene",stringsAsFactors=FALSE,header=TRUE)
>targets<-readTargets(file="targets.txt")
>Group<-factor(paste(targets$Condition))
>cbind(targets,Group=Group)
```

Build the generalized linear model that will be used for differential expression testing

```
>dge<-DGEList(counts=x,group=Group)
>keep<-filterByExpr(dge)
>table(keep)
>dge<-dge[keep,keep.lib.sizes=FALSE]
>cpm<-cpm(dge)
>lcpm<-cpm(dge,log=TRUE)
```

Normalization

```
>dge_norm<-calcNormFactors(dge)
```

MDS plot

```
>par(mfrow=c(1,1))
>col.cell<-
c("blue","blue","blue","purple","purple","purple","red","red","red","black","bl
ack","black")
>plotMDS(dge_norm,col=col.cell)
```

Set study design

```
design<-model.matrix(~0+Group,data=dge_norm$samples)
>colnames(design)<-levels(dge_norm$samples$group)
```

Estimate dispersion

```
>dge_Disp<-estimateDisp(dge_norm,design,robust=TRUE)
```

Set contrasts

```
>my.contrasts<-makeContrasts(RES=PCB126-RES,
                RES_AFB1 = RES_AFB1-AFB1,
                Levels = design)
```

Quasi-likelihood F-tests

```
>fit<-glmQLFit(dge_Disp,design,robust=TRUE)
>glm.RES<-glmQLFTest(fit,contrast=my.contrasts[,"RES"])
>TopTags_RES<-topTags(glm.RES,
                N = Inf,
                adjust.method = "fdr",
                sort.by = "PValue")
>write.table(file="glm_RES.txt",TopTags_RES,sep='\t',quote=F,row.names=T)
>is.de<-decideTestsDGE(glm.RES)
>summary(is.de)
>glm.RES_AFB1<-glmTreat(fit,contrast=my.contrasts[,"RES_AFB1"])
```

```
>TopTags_RES_AFB1<-
topTags(glm.RES_AFB1,n=Inf,adjust.method="fdr",sort.by="PValue")
>write.table(file="glm_RES_AFB1.txt",TopTags_RES_AFB1,sep='\t',quote=F,row.name
s=T)
>is.de<-decideTestsDGE(glm.RES_AFB1)
>summary(is.de)
```

➔ *DEGs HEATMAP*

```
>x<-read.delim("input_Log10CPM_top10.txt",
               row.names = "Gene",
               stringsAsFactors = FALSE,
               header = TRUE)
>head(x)
>library("pheatmap")
>library("RColorBrewer")
>data<-x
>head(data)
>basedir<-getwd()
>maxclust<-4
>col.pal<-brewer.pal(11,"RdYlGn")
>drows1<-"euclidean"
>dcols1<-"euclidean"
>filename<-"my.pheatmap.tiff"
>outfile<-paste(basedir,filename,sep="/")
>hm.parameters<-list(data,
               color = col.pal,
               cellwidth = 15, cellheight = 12, scale = "none",
               treeheight_row = 200,
               kmeans_k = NA,
               show_rownames = T, show_colnames = T,
               main = "Top DEGs",
               clustering_method = "average",
               cluster_rows = FALSE, cluster_cols = TRUE,
               clustering_distance_rows = drows1,
               clustering_distance_cols = dcols1,
               width=12, heigh=13)
>do.call("pheatmap", c(hm.parameters, filename=outfile))
```

➔ *FUNCTIONAL ANALYSIS (ClusterProfiler)*

```
>library(clusterProfiler)
>library(enrichplot)
>library(org.Bt.eg.db)
>library(pathview)
>library(DOSE)
>library(ggplot2)
>library(viridis)
```

1. *Effects of curcuminoids on AFB1-induced transcriptional changes (R+AFB1 vs AFB1)*

1.1 Data input (DEGs and logFC)

```
>RES<-read.csv("DEGS_RES_AFB1.csv",sep=";")
>geneList<-RES[,2]
```

```
>names(geneList)<-as.character(RES[,1])
>geneList<-sort(geneList,decreasing=TRUE)
>DEGs<-names(geneList)
```

1.2 Set a background (expressed genes)

```
>back<-topTags(glm.RES_AFB1,n=Inf)$table
>back_list<-row.names(back)
```

1.3 GO over-representation test

```
>enrichBP_DEGS_RES<-enrichGO(gene=DEGs,
                OrgDb = org.Bt.eg.db,
                keyType = "ENSEMBL",
                ont = "BP",
                pvalueCutoff = 0.05,
                pAdjustMethod = "BH",
                universe = back_list,
                qvalueCutoff = 0.2,
                minGSSize = 2,
                maxGSSize = 500,
                readable = TRUE,
                pool = FALSE)
>enrichBP_DEGS_RES_simply<-simplify(enrichBP_DEGS_RES,
                Cutoff = 0.5,
                By = "p.adjust",
                select_fun = min)
>dotplot(enrichBP_DEGS_RES_simply, showCategory=20)
```

1.4 Convert ENSEMBL gene IDs to gene names

```
>enrichBP_Gene_DEGS_RES_AFB1_simply<-
>setReadable(enrichBP_DEGS_RES_AFB1_simply,"org.Bt.eg.db",keyType="ENSEMBL")
```

1.5 Convert ENSEMBL IDs to ENTREZ IDs

```
>DEGS_ENTREZ<-
>bitr(DEGs,fromType="ENSEMBL",toType=c("ENTREZID"),OrgDb=org.Bt.eg.db)
>DEGS_ENTREZ_OK<-DEGS_ENTREZ$ENTREZ
>back_ENTREZ<-bitr(back_list,fromType="ENSEMBL",
                toType = c("ENTREZID"),
                OrgDb = org.Bt.eg.db)
>Back_ENTREZ_OK<-back_ENTREZ$ENTREZID
```

1.6 KEGG over-representation test

```
>KEGG_DEGS_RES_AFB1<-enrichKEGG(gene=DEGs_ENTREZ_OK,
                Organism = "bta",
                pvalueCutoff = 0.05,
                pAdjustMethod = "BH",
                universe = Back_ENTREZ_OK,
                minGSSize = 5,
                maxGSSize = 500,
                use_internal_data = FALSE)
>KEGG_DEGS_RES_AFB1
>dotplot(KEGG_DEGS_RES_AFB1, showCategory=20)
```

1.7 KEGG Gene Set Enrichment Analysis

```
>inputGSEA_RES_AFB1<-read.csv("input_GSEA_RES_AFB1.csv",sep=";")
```

```
>geneList_GSEA<-inputGSEA_RES_AFB1[,2]
>names(geneList_GSEA)<-as.character(inputGSEA_RES_AFB1[,1])
>geneList_GSEA<-sort(geneList_GSEA,decreasing=TRUE)
>kegg<-gseKEGG(geneList = geneList_GSEA,
                organism = "bta",
                minGSSize = 3,
                maxGSSize = 800,
                pvalueCutoff = 0.05,
                pAdjustMethod = "BH",
                keyType = "ncbi-geneid")

>ridgeplot(kegg)+labs(x="enrichment distribution")
```
