# Supplementary material for: Discovering the Protective Effects of Resveratrol on Aflatoxin B1-Induced Toxicity: A Whole Transcriptomic Study in a Bovine Hepatocyte Cell Line
Source: Antioxidants (Basel). 2021 Jul 29;10(8):1225. doi: 10.3390/antiox10081225 (PMC8388899; doi:10.3390/antiox10081225)
Supplement: Supplementary file 1 [file antioxidants-10-01225-s001.zip › antioxidants-1308402-supplementary/Supplementary_rev/TableS1.pdf]

Table S1. Sequencing and mapping results. The table reports the RNA-seq libraries sequenced, including for each of them: i) the number of raw reads obtained; ii) the number of reads after trimming and rRNAs removal; iii) the number of mapped reads (and the percentage of mapped reads).

Sample ID	N° raw reads	N° reads after trimming and rRNAs removal	N° of reads mapping (%)
PCB126_B	22,928,709	22,823,199	22,628,132 (99.15)
PCB126_C	25,807,296	25,653,873	25,436,649 (99.15)
PCB126_D	43,005,307	42,793,203	42,432,959 (99.16)
AFB1_B	20,399,647	20,286,542	20,106,412 (99.11)
AFB1_C	23,877,815	23,632,276	23,428,800 (99.14)
AFB1_D	10,774,628	10,714,111	10,614,084 (99.07)
R_B	36,914,107	36,757,939	36,454,028 (99.17)
R_C	30,670,475	30,529,140	30,268,391 (99.15)
R_D	14,743,375	14,647,652	14,522,588 (99.15)
R+AFB1_B	17,072,624	17,002,499	16,854,014 (99.13)
R+AFB1_C	20,829,097	20,737,398	20,579,187 (99.24)
R+AFB1_D	24,813,275	24,592,089	24,362,943 (99.07)
