# Supplementary figures and images for: Discovering the Protective Effects of Resveratrol on Aflatoxin B1-Induced Toxicity: A Whole Transcriptomic Study in a Bovine Hepatocyte Cell Line
Source: Antioxidants (Basel). 2021 Jul 29;10(8):1225. doi: 10.3390/antiox10081225 (PMC8388899; doi:10.3390/antiox10081225)

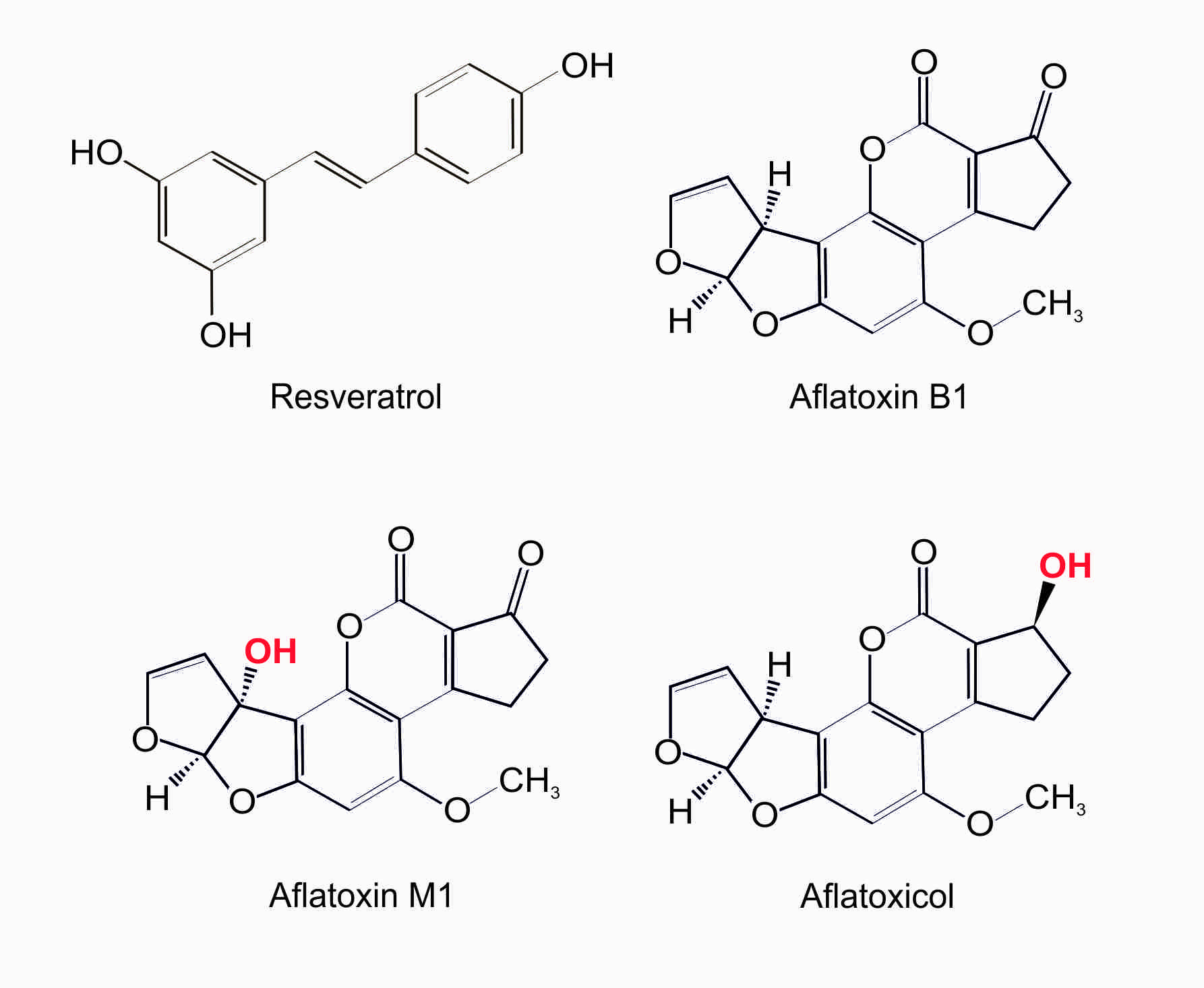

Supplement: Supplementary file 1 [file antioxidants-10-01225-s001.zip › antioxidants-1308402-supplementary/Supplementary_rev/FigureS1_rev.jpg]

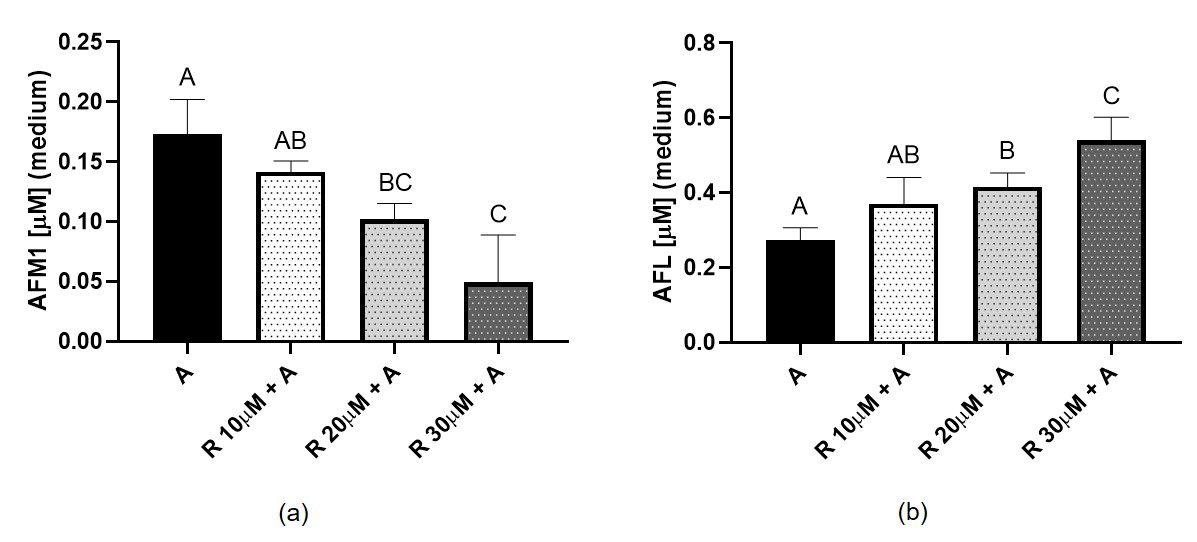

Supplement: Supplementary file 1 [file antioxidants-10-01225-s001.zip › antioxidants-1308402-supplementary/Supplementary_rev/FigureS2_rev.jpg]

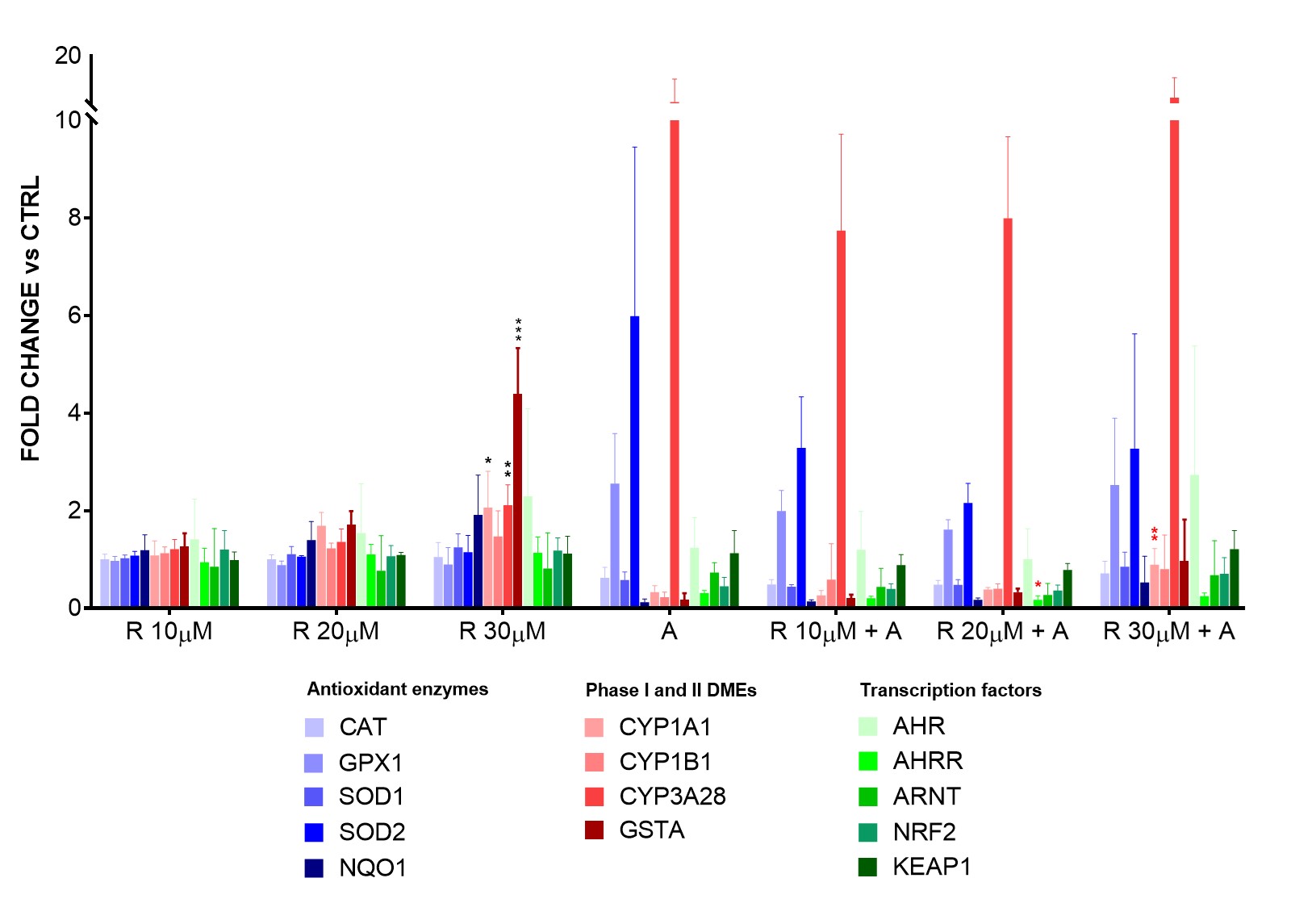

Supplement: Supplementary file 1 [file antioxidants-10-01225-s001.zip › antioxidants-1308402-supplementary/Supplementary_rev/FigureS3_rev.jpg]

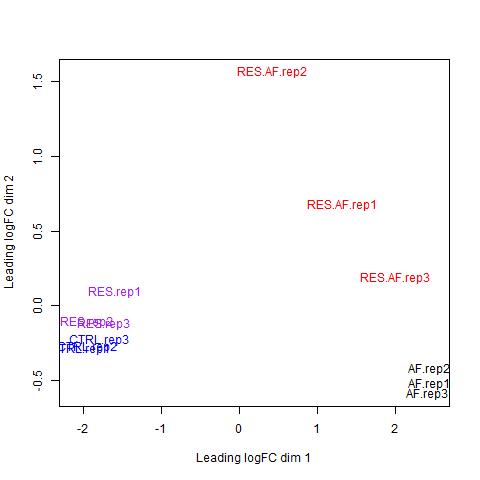

Supplement: Supplementary file 1 [file antioxidants-10-01225-s001.zip › antioxidants-1308402-supplementary/Supplementary_rev/FigureS4_rev.jpg]

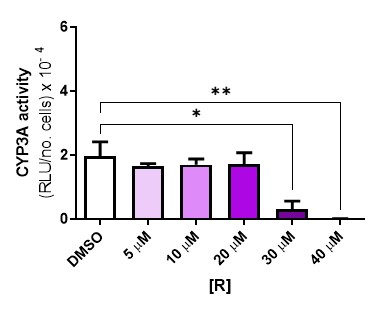

Supplement: Supplementary file 1 [file antioxidants-10-01225-s001.zip › antioxidants-1308402-supplementary/Supplementary_rev/FigureS5_rev.jpg]
